# Use of Rapid Drug Desensitization in Delayed Hypersensitivity Reactions to Chemotherapy and Monoclonal Antibodies

**DOI:** 10.3389/falgy.2021.786863

**Published:** 2022-01-14

**Authors:** Arantza Vega, M. Isabel Peña, Inés Torrado

**Affiliations:** ^1^Department of Allergy, University Hospital of Guadalajara, Guadalajara, Spain; ^2^ARADyAL Spanish Thematic Network and Co-operative Research Centre RD16/0006/0023, Instituto de Salud Carlos III (ISCIII), Fundación Española para la Ciencia y la Tecnología (FECyT), Madrid, Spain

**Keywords:** rapid drug desensitization, desensitization, chemotherapeutic drugs, monoclonal antibodies, delayed drug reactions, no-immediate drug reactions

## Abstract

**Background:**

Rapid drug desensitization (RDD) allows first-line therapies in patients with immediate drug hypersensitivity reactions (DHR) to chemotherapeutic drugs (ChD) and monoclonal antibodies (mAb). Desensitization in delayed drug reactions has traditionally used slow protocols extending up to several weeks; RDD protocols have been scarcely reported.

**Patients and Method:**

We retrospectively analyzed the patients referred to the Allergy Department, who had experienced a delayed DHR (> 6 h) related to a ChD or mAb and underwent an RDD protocol. The rate of successful administration of the offending drug and the presence of adverse reactions were evaluated.

**Results:**

A total of 93 RDDs were performed in 11 patients (including 6 men and 5 women, with a median age of 61 years). The primary DHR were maculopapular exanthema (MPE) (8), generalized delayed urticaria (1), MPE with pustulosis and facial edema (1), and facial edema with desquamative eczema (1). The meantime for the onset of symptoms was 3 days (range 1–16 days). RDD was performed using a protocol involving 8–13 steps, with temozolomide (25), bendamustine (4), rituximab (9), infliximab (24), gemcitabine (23), and docetaxel (8), within 4.6–6.5 h. Sixteen breakthrough reactions were reported during the RDD (17.2 %) in 5 patients; all were mild reactions including 11 delayed and 5 immediate reactions. All patients completed their treatment.

**Conclusions:**

RDD is a potentially safe and effective procedure in patients suffering from delayed reactions to ChD and mAb. It allows them to receive full treatment in a short period, thereby reducing time and hospital visits.

## Introduction

Rapid drug desensitization (RDD) has shown to be a safe and effective procedure for patients presenting with an immediate drug hypersensitivity reaction (DHR) (1). Chemotherapeutic drugs (ChD) and monoclonal antibodies (mAb) have been administered by means of this procedure for many years, allowing patients to get their first-line therapies (2–4). Several protocols have been proposed and some modifications have been applied (5–7). However, the one established at the Brigham and Women's Hospital, which is a three-solution, 12-step protocol, has been widely accepted (8, 9). The drug is delivered in consecutive steps at increasing doses by raising the infusion rate and the solution concentration, at fixed 15 min intervals, with the exception of the last step, in a total time of 5–6 h. Dose increments are 2–2.5 times that of the previous step. The number of solutions and steps can be modified depending on the patient's stratification risk and RDD tolerance.

Traditionally, delayed drug reactions have been treated with slow desensitization procedures that could extend for days or even weeks (10–14). There is a broad literature describing RDD for delayed reactions, including co-trimoxazole, betalactam antibiotics, sulfonamides and other antibiotics, such as ciprofloxacin, clindamycin, and tetracyclines, among others (15–18). However, there is little experience, with less extensive and more controversial literature, on RDD for chemotherapeutic agents and monoclonal antibodies (2, 19), although the European Academy of Allergy and Clinical Immunology (EAACI) position paper of the Drug Allergy Interest Group recommends performing an RDD on immediate DHR and on uncomplicated and non-serious, mild delayed drug reactions (20).

This study describes the use of RDDs with chemotherapeutic agents and monoclonal antibodies in patients with delayed DHR.

## Patients and Method

A retrospective study was performed on patients who received desensitization as part of the standard care at the Allergy Department of University Hospital of Guadalajara, Spain. The study protocol was approved by the local ethics committee.

All patients had suffered a delayed hypersensitivity reaction related to ChD or mAb administration. The primary endpoints were the rate of successful administration of the offending drug and the presence of breakthrough reactions (BTR) using a rapid desensitization protocol.

### Patients

This is a single institution-based retrospective study. Patients referred to the Allergy Department, who fulfilled the following criteria: (1) those who experienced DHR related to a ChD or mAb; (2) symptoms that started more than 6 h after drug administration; (3) those who needed to take the treatment with the same drug; and (4) those who accepted to undergo an RDD and signed informed consent.

Patients with severe delayed reactions, such as serum sickness, skin reactions with eosinophilia and systemic symptoms, Stevens–Johnson syndrome, or toxic epidermal necrolysis were excluded.

### Allergological Study

Signs and symptoms of the hypersensitivity reaction were recorded. Skin prick tests with ChD and mAb were run at full strength of the drug, and if negative, were followed by intradermal tests using serial dilutions (21) with non-irritating concentrations. Results were interpreted in 15–20 mins, and after 24 and 72 h (19, 22). Serum saline and histamine were used as negative and positive controls, respectively. Patch tests were run when the parenteral form of the drug was not available. Hypersensitivity to other drugs was ruled out with skin and challenge tests, if there was a concomitant administration and the patients had not subsequently received the drug (23).

### Desensitization Protocol

Depending on the administered drug, the patients were pretreated 20–30 min before the desensitization protocol with corticoids, antiemetics, antihistamines, or acetaminophen, as dictated by the oncology standards or product information.

A standard 12-step protocol described by Brigham and Women's Hospital group was used with the intravenous drugs (4, 8, 24, 25). For oral drugs, a 13-step protocol adapted from the previous one was elaborated (26). The total time for desensitization ranged from 4 to 6.5 h.

All desensitizations were carried out in an outpatient basis at the allergy day care unit, and performed by allergists and specially trained nurses. Resuscitation personnel and resources were easily available. Beta-blockers were held for 24 h before desensitization.

### Breakthrough Reactions During and After Desensitization

If an adverse reaction appeared during desensitization, the drug infusion was immediately stopped. Depending on the patient's symptoms (27), if required, treatment with intramuscular epinephrine, intravenous dexchlorpheniramine, hydrocortisone or 6-metilprednisolone, acetaminophen, ondansetron, or nebulized salbutamol was available. Once the reaction subsided, the protocol was restarted from the step at which it had been paused (4, 8). If a delayed reaction took place, the patient was evaluated and treated at emergency department of the hospital and reevaluated at the allergy department before the next desensitization.

Depending on the immediate and delayed tolerance to the RDD, the protocol was modified in subsequent desensitizations by adding or reducing steps and/or administering prophylactic medication before and/or after the RDD (4, 28).

### Statistical Analysis

The analysis was descriptive and no hypothesis tests were performed. Quantitative variables were described as the mean and standard deviation (SD) or as medians and interquartile range (IQR, defined as percentiles 25 and 75), whereas categorical variables were presented as frequency and percentage. All analyses were performed using the SPSS package (IBM SPSS Statistics for Windows, Version 20.0. Armonk, NY: IBM Corp).

## Results

### Characteristics of the Patients and the Initial Reactions

A total of 11 patients who fulfilled the inclusion criteria of the study, including 6 men and 5 women with a median age of 61 years (IQR 44.5–68.5 years), were admitted in the desensitization unit between January 2009 and March 2021. Nine patients were receiving oncologic treatment, while the other 2 were treated for Crohn's disease and ankylosing spondylitis, respectively. Table 1 summarizes the demographic and clinical characteristics of the patients in the study. Only one patient was atopic and 2 had been previously diagnosed with hypersensitivity to other drugs.

**Table 1:** Patients characteristics.

**Patients**					**Drug hypersensitivity reactions**						**Rapid drug desensitization**				
**ID No**	**Gender**	**Age**	**Allergy**	**Diagnosis**	**Drug**	**DHR**	**Time interval (days)**	**Dose No**	**Repeated?**	**Skin test**	**Protocol steps**	**RDD No**	**Delayed BTR**	**Immediate BTR**	**Protocol change**
1	F	33	Respiratory	Brain cancer	Temozolomide	MPE	3.5	5	Yes	N	13	15	0	0	No
2	F	65	No	Brain cancer	Temozolomide	MPE	9	7	No	ND	13	10	2 (1st, 2nd)	3 (3rd, 4th, 5th)	No
3	M	70	Drug	Non-Hodgkin Lymphoma	Rituximab	MPE	1	1	Yes	P	12	9	1 (1st)	2 (2nd, 3rd)	Cetirizine and Prednisone post RDD 2 days
4	F	38	No	Spondylitis	Infliximab	MPE	1	2	Yes	N	12	5	0	0	Decrease steps
5	M	48	No	Crohn disease	Infliximab	Delayed urticaria	4	3	Yes	N	12	19	0	0	No
6	M	61	No	Urothelial cancer	Gemcitabine	MPE	3	1	Yes	N	12	10	0	0	Increase final rate
7	M	74	No	Lung cancer	Gemcitabine	MPE	2	1	Yes	N	12	3	0	0	No
8	M	41	No	Lung cancer	Gemcitabine	MPE + pustulosis	2	1	Yes	N	12	7	5 (1^st^-5th)	0	No
9	F	59	No	Urothelial cancer	Gemcitabine	MPE	1	2	Yes	N	8	3	2 (2nd, 3rd)	0	Prednisone post RDD 3 days
10	F	68	Drug	CLL	Bendamustine	MPE	16	1	No	N	12	4	1 (1st)	0	Prednisone post RDD 3 days
11	M	69	No	Prostate cancer	Docetaxel	Desquamative facial edema	5	2	No	P	12	8	0	0	No

*ID No., identification number; Gender: F, female; M, male; DHR, drug hypersensitivity reaction; Time interval, elapsed time from the drug administration and the appearance of symptoms; Dose No., Drug dose that elicited the HSR; Repeated?, if the HSR occurred more than once before the allergological evaluation; Protocol steps, steps of the desensitization protocol used for RDD; RDD No., number of rapid drug desensitizations carried out; BTR, breakthrough reactions; immediate BTR, reactions during the RDD; Delayed BTR, breakthrough reactionsthat started >6 h after the RDD; Immediate and Delayed BTR between brackets, the number of the desensitization when the BTR took place; CCL, chronic lymphocitic leukemia; MPE, maculopapular exanthema; Protocol change, changes of desensitization protocol in successive administrations*.

The initial DHRs included 8 cases (6 of them had associated eyelid angioedema) of maculopapular exanthema (MPE); one patient had generalized delayed urticaria with facial angioedema; another presented MPE with pustulosis and facial edema (acute generalized exanthematous pustulosis was ruled out by Dermatology); and another showed facial edema with desquamative eczema.

Eight patients had presented the same reaction with the offending drug in two or more consecutive administrations before being referred for an allergy evaluation.

Implied drugs were temozolomide (2), bendamustine, rituximab, infliximab (2), gemcitabine (4), and docetaxel. The mean time between the drug administration and the onset of the DHR was 3 days, with an average between 1 to 16 days, and a mean time of 3 days (IQR 1.5–4.5).

### Allergological Study

Skin tests were performed on 10 patients, in which 9 patients underwent prick and intradermal tests and one patient underwent patch test with temozolomide. Drugs and concentrations used are detailed in Table 2. Positive intradermal tests were obtained in 2 patients (one immediate reading for rituximab and one 24 h delayed reading for docetaxel). At the time when the allergological study was performed, the patients with ID Numbers 1, 2, 5, 8, and 11 were on steroid treatment.

**Table 2:** Drug concentration for skin testing.

**Drug**	**Prick (mg/ml)**	**Intradermal (mg/ml)**	**Patch test**
Temozolomide	NU	NU	5%*
Bendamustine	2,5	0.025–0.25	
Gemcitabine	1	0.01–1	
Docetaxel	10	0.01–1	
Rituximab	10	1–10	
Infliximab	10	1–10	

*Drug concentrations used in skin test. NU, not used due to the lack of parenteral drug at the moment of performing skin tests.*

*Results were interpreted at 15–20 mins and after 24–48 h.*

*Patch test were performed at 5% both in aqueous solution and in petrolatum.

Hypersensitivity to concomitantly administered drugs was ruled out. In some cases, the patients had tolerated them before the allergy evaluation (dexamethasone, carboplatine, ondasentron, metroclopramide, and difenhydramine); while in 4 patients, the skin and drug challenge tests were carried out with negative results (alopurinol, ondansetron, cotrimoxazole, acyclovir, levofloxacin, and erythropoietin).

The diagnosis of drug hypersensitivity was established by the recurrence of DHR in 2 or more successive administrations with the same drug in 7 patients and by skin testing in 2 other patients. In the case of the two remaining patients, there was suspicion about the implied drug, which was later confirmed when both of them experienced BTR reactions during RDD.

### Rapid Drug Desensitizations

A total of 93 RDDs were performed in the 11 patients, which included 25 with temozolomide, 4 with bendamustine, 9 with rituximab, 24 with infliximab, 23 with gemcitabine, and 8 with docetaxel. Nine patients received pretreatment before RDD, prescribed by an oncologist or the patient's specialist, which involved metoclopramide or dexamethasone and ondansetron with gemcitabine; 6-metyl-prednisolone and acetaminophen with infliximab; 6-metyl-prednisolone and diphenhydramine with rituximab; dexamethasone, diphenhydramine, ranitidine, acetaminophen, and ondansetron with bendamustine; dexamethasone, diphenhydramine, and ranitidine with docetaxel. Additionally, we added diphenhydramine in the treatment of two patients receiving Infliximab (patient No. 4 and 5), and added deflazacort and cetirizine to a third patient (Patient No. 2).

The RDD was performed intravenously in 9 patients, with a 12-step protocol in 8 patients (63 RDDs) lasting around 4.6–5.6 h depending on the final infusion rate, and an 8-step protocol in 2 patients (5 RDDs) lasting 4 h, while 2 patients received a 13-step oral protocol (25 RDDs) lasting 6.5 h. The first desensitization was performed in all the patients in <4 weeks after the delayed hypersensitivity reaction.

### Breakthrough Reactions During and After RDD

Five patients experienced a total number of 16 hypersensitivity reactions during desensitization (17.2% of the total RDDs). They included 11 delayed reactions in 5 patients: 5 isolated pustulosis lesions with macular exanthema, 3 macular exanthemas, 1 MPE, and 2 delayed pruritus. All of them occurred after the first course of RDD (Table 3). Five immediate reactions were observed in 2 patients: 5 macular exanthemas, with dyspnea and oxygen desaturation, observed in one of them. Both patients had previously experienced delayed BTR with former RDDs. All delayed reactions subsided, with five subsiding spontaneously and 6 with the use of corticoids. Immediate reactions were subsided completely. They were handled at the allergy day care unit, one with corticoids and salbutamol, while the others subsided spontaneously.

**Table 3:** Characteristics of breakthrough reactions during RDD.

**ID No**	**Drug**	**DHR**	**Total RDD**	**BTR**	**RDD No**	**Time**	**Symptoms**	**Protocol change**
2	Temozolomide	MPE	10	5	1	24 h	MPE	No
					2	24 h	MPE	No
					3	Steps 10 and 11	MPE	No
					4	Step 11	MPE	No
					5	Step 10	MPE	No
3	Rituximab	MPE	9	3	1	2 days	ME	Cetirizine and Prednisone post RDD 2 days
					2	Step 12	ME, dyspnea and O_2_ desaturation	
					3	Step 11	ME	
8	Gemcitabine	MPE + pustulosis	7	5	1	3–4 days	Isolated	No
					2		pustulosis	
					3		lesions	
					4		with	
					5		macular exanthema	
9	Gemcitabine	MPE	3	2	2	2 days	Pruritus	Prednisone post RDD 3 days
					3	6 h	Pruritus	
10	Bendamustine	MPE	4	1	1	6 days	MPE	Prednisone post RDD 3 days

*ID No., identification number; DHR, drug hypersensitivity reaction; Total RDD, number of rapid drug desensitizations carried out in each patient; BTR, reactions during the RDD; RDD No., number of desensitization were the BTR took place; Time, moment of symptoms onset: step during the infusion or time after the end of the drug administration; MPE, maculopapular exanthema; ME, macular exanthema; protocol change, changes of desensitization protocol in successive administrations*.

In patients who presented several reactions, the time interval for the onset of symptoms decreased with every desensitization, and so was the duration and severity (Table 3).

Three patients who suffered adverse delayed reactions were pretreated with prednisone after the RDD in some of the subsequent administrations. On the contrary, in some patients who showed a good tolerance to the desensitization, the protocol was modified to reduce the total time. All patients received the full target dose and all the administrations prescribed by their specialists.

## Discussion

We present the results of 93 RDDs with ChD and mAb carried out in 11 patients who had suffered delayed, uncomplicated, and non-serious reactions with these drugs. In the initial DHR, the meantime from the onset of symptoms was 3 days; two patients experienced an interval longer than a week. In published large series, RDDs are performed only in immediate reactions (5, 29) or in delayed reactions starting within the first 48 h after drug administration (4–6). RDD has also shown effectiveness in delayed drug reactions to ChD and mAb in short series or case reports (26, 30–33).

Most of our patients had suffered 2 DHR with the same drug before being referred for an allergy evaluation. This shows the lack of suspicion when dealing with delayed reactions, especially when the interval of symptoms is longer than 3 days. Delayed cutaneous reactions to drugs generally become apparent 4–21 days after exposure, with reactions reaching their maximum after 24–72 h. Subsequent reactions may develop within 1 or 2 days, with initial symptoms appearing after only a few hours (34). This was observed in one of our patients, who presented a DHR 16 days after receiving bendamustine and developed a BTR with the first desensitization, around 6 days later. Delayed reactions may well appear after only 1 day or even earlier in case of presensitization or direct interaction of the drug with the receptor (pi-concept) (35).

Cutaneous tests were not of value for diagnosis in our study, as opposed to other authors (2, 4). Only 2 patients showed a positive result. The sensitivity and specificity of delayed intradermal skin test readings have not been established. The tests were helpful in the diagnosis of non-severe delayed reactions induced by beta-lactam antibiotics, radiocontrast media, heparins, and biological agents (23). Moreover, some authors did not perform skin tests with certain drugs because validation of these clinical tests does not exist or the negative predictive value is unknown (4, 36). It is also important to remark that half of the patients were under a chronic corticoid treatment that could not be suspended. They were already receiving it when they suffered the DHR. Even though the corticosteroids did not avoid the DHR, they could influence the results of the allergological study. This could lead to false-negative results in the delayed reading of skin tests. Moreover, the recommendation to perform the allergological study 4–6 weeks after the hypersensitivity reaction had subsided (19, 37) appeared to be not possible in most of our patients due to the characteristics of some reactions, lasting for several days or weeks, and the patients' need for urgent treatment which could not be postponed. In clinical practice, it is difficult to wait for the recommended period to perform skin tests for ChD due to the oncologic strict administration schemes that must not be changed to preserve efficacy and avoid toxicity (38). So, it is important to have in mind that a false-negative result is possible (23). Even so, the diagnosis of delayed DHR was well established in our patients: 8 patients experienced the same reaction to the same drug at least twice (one of these cases also had a positive skin test); another patient showed a positive intradermal test; and the remaining 2 suffered from BTR during the RDD. Some patients were in need of skin and challenge tests to rule out hypersensitivity to other concomitant drugs, as previously described (28, 39, 40).

Desensitization procedures for delayed drug reactions have been traditionally treated with slow desensitization protocols lasting over many days or even weeks (10–14). The introduction of rapid protocols represents a better approach to achieve therapeutic doses, thereby reducing patient visits and resources to a few hours, and maintaining patients on their most effective treatments (3, 4, 41).

In published series breakthrough reactions during RDD to ChD and mAB are up to 25% (2, 4–6, 19, 29). In our study, BTR were present in 17% of cases, i.e., in 5 patients. The BTR were initially delayed and they took place after the first desensitization, in concordance with previous publications (6, 8, 29). We want to emphasize how the time-to-onset of BTR was shorter with the subsequent desensitization procedures, and became immediate in the last steps of the RDD in 2 patients. BTR severity also decreased. In a sensitized individual, the initial symptoms with subsequent reactions could appear within few hours (6, 34). Some studies also report the change from a delayed to an immediate DHR. In one such study, Picard et al. describe that among patients with hypersensitivity to taxanes, 4 patients with an initial delayed DHR had a recurrent reaction that was immediate with RDD (19). Jiménez-Rodríguez described the “converter phenotype” in patients treated with taxanes, who presented delayed DHR and in subsequent exposures developed immediate HSR, generally type I reactions (42). Other authors suggest that type IV reactions may predispose the development of IgE-mediated type I reactions (2). All reactions related to the desensitization, both immediate and delayed, were easily solved. Epinephrine was not used. RDD was a safe procedure in these patients when performed by a trained allergy team. As shown in Table 3, we continued with a corticosteroid treatment after the RDD, for 3 days in 3 patients. This treatment did not prevent the appearance of new reactions in 2 of them. There is no clear evidence about the use of premedication before or after RDD (20, 28) and there is also a considerable variation regarding diagnostic procedures and therapeutic approaches (43).

This study has some limitations. There are a variety of diseases and drugs implied in the DHR in our patients. The allergological study is difficult in oncologic patients with DHR due to the short time available to perform the skin tests, the treatment with corticoids in many of them, and the lack of validation of skin test for delayed reactions with these drugs.

Drug desensitization is an effective and safe option that allows patients with DHR to continue their first-line treatments. It has proved to be a safe procedure when performed by experienced allergists. It has no additional costs and survival outcomes with no differences from those via standard administration (3, 4, 43, 44).

RDD in delayed reactions can achieve the target dose in a short time, which is a matter of great importance in this type of patients. The RDD used in our patients is a flexible protocol that can be lengthened or shortened depending on their tolerance (4, 8, 19, 28, 45). It has allowed to receive full treatment to all of them.

## Conclusions

Rapid drug desensitization could be a safe and effective procedure for patients suffering from delayed reactions to ChD and mAb, allowing them to continue the optimal treatment for their diseases. Full target doses are achieved in a few hours, reducing patients' visits to the hospital.

## Data Availability Statement

The original contributions presented in the study are included in the article/supplementary material, further inquiries can be directed to the corresponding author/s.

## Ethics Statement

The studies involving human participants were reviewed and approved by Ethics Committee of the University Hospital of Guadalajara. Written informed consent for participation was not required for this study in accordance with the national legislation and the institutional requirements.

## Author Contributions

AV designed the study. All authors have participated in the review, written, and approved the manuscript.

## Funding

Part of this work was supported by the Instituto de Salud Carlos III (ISCIII) co-funded by Fondo Europeo de Desarrollo Regional—FEDER for the Thematic Networks and Co-operative Research Centres: ARADyAL (RD16/0006/0023).

## Conflict of Interest

The authors declare that the research was conducted in the absence of any commercial or financial relationships that could be construed as a potential conflict of interest.

## Publisher's Note

All claims expressed in this article are solely those of the authors and do not necessarily represent those of their affiliated organizations, or those of the publisher, the editors and the reviewers. Any product that may be evaluated in this article, or claim that may be made by its manufacturer, is not guaranteed or endorsed by the publisher.

## Previous Presentation

Part of the data included in this manuscript was presented in oral communication format during the Spanish Society of Allergy and Clinical Immunology (SEAIC) Annual Congress in 2017.

## Abbreviations

BTR: breakthrough reactions
ChD: chemotherapeutic drugs
DHR: drug hypersensitivity reactions
mAb: monoclonal antibodies
MPE: maculopapular exanthema
RDD: rapid drug desensitization.
